# Functional positioning in robotic patello-femoral arthroplasty: a step-by-step technique

**DOI:** 10.1051/sicotj/2025029

**Published:** 2025-06-11

**Authors:** Luca Andriollo, Christos Koutserimpas, Pietro Gregori, Jean Baltzer, Elvire Servien, Cécile Batailler, Sébastien Lustig

**Affiliations:** 1 Orthopaedics Surgery and Sports Medicine Department, FIFA Medical Center of Excellence, Croix-Rousse Hospital, Hospices Civils de Lyon, Lyon North University Hospital 103 Grande Rue de la Croix-Rousse 69004 Lyon France; 2 Ortopedia e Traumatologia, Fondazione Poliambulanza Istituto Ospedaliero 25124 Brescia Italy; 3 School of Rehabilitation Health Sciences, University of Patras 26504 Greece; 4 Fondazione Policlinico Universitario Campus Bio-Medico 00128 Roma Italy; 5 LIBM-EA 7424, Interuniversity Laboratory of Biology of Mobility, Claude Bernard Lyon 1 University 69100 Lyon France; 6 Univ Lyon, Claude Bernard Lyon 1 University, IFSTTAR, LBMC UMR_T9406 69622 Lyon France

**Keywords:** Functional positioning, Personalized knee arthroplasty, Robotic knee, Patello-femoral arthroplasty, PFA

## Abstract

Patello-femoral arthroplasty (PFA) is an effective treatment option for isolated patello-femoral osteoarthritis. However, challenges remain regarding implant positioning and patellar tracking. Recent advances in implant design and robotic-assisted techniques have contributed to more personalized and reproducible procedures. Functional positioning (FP), a three-dimensional alignment concept, introduces a customized approach to optimize trochlear resurfacing and restore joint kinematics of the anterior compartment. This article presents a step-by-step surgical technique for PFA using FP principles in combination with an image-based robotic system. The technique ensures accurate preoperative planning, real-time intraoperative adjustments, and precise component placement. The key steps of this surgical technique include trochlear resurfacing assisted by an image-based robotic system and the restoration of patellar tracking, following a step-by-step approach that is both effective and reproducible. The use of FP enables personalized anterior compartment restoration, avoiding overstuffing and improving patellar tracking. Future studies will help refine FP strategies and further optimize outcomes in these patients.

## Introduction

Isolated patella-femoral osteoarthritis (PF-OA) is a relatively common condition, particularly in older adults. It affects around 10% of individuals over the age of 40, with the prevalence increasing to about 24% in women and 11% in men over 55 years of age [1, 2]. Patello-femoral arthroplasty (PFA) represents a promising and effective treatment option for patients with isolated PF-OA, particularly when the ligamentous structures of the knee are preserved [3]. It provides pain relief and functional improvement while delaying or avoiding the need for total knee arthroplasty (TKA) [2].

In recent years, advancements in implant technology have shown significant potential in addressing previous limitations, resulting in better functional outcomes and fewer mechanical complications [3, 4]. This has been further supported by the introduction of second-generation inlay implants, specifically designed to anatomically resurface the trochlea. Moreover, the development of robotic-assisted knee arthroplasty has contributed to more reproducible, accurate, and personalized implant positioning [5–7]. It is becoming increasingly important to consider not only the patient’s bony anatomy but also joint kinematics and soft tissue compliance [8–10].

This surgical technique, referred to as functional positioning (FP), describes a personalized approach to PFA that aims to preserve joint anatomy and kinematics using an image-based robotic system.

## Surgical technique

The surgical technique is demonstrated in Video 1. Indications and contraindications have been previously described in detail by Batailler et al. [11]. The patient is positioned in a standard supine position, with one arm placed on a lateral support in abduction and the other resting on the surgical table. A lateral pad is positioned centered on the thigh, while a distal pad is used to maintain the knee at a 90° angle during the procedure.

### Step 1: Pre-operative planning

Pre-operative planning refers to the phase of planning for the positioning of the prosthetic implant, specifically a PFA (RESTORIS MCK^®^ partial knee implant system, Stryker^®^, Mahwah, USA). The planning is carried out using a specific computer navigation tool (Orthomap ASM^®^, Stryker^®^, Mahwah, USA), after performing a CT scan and generating a 3D model.

**Figure 1:**
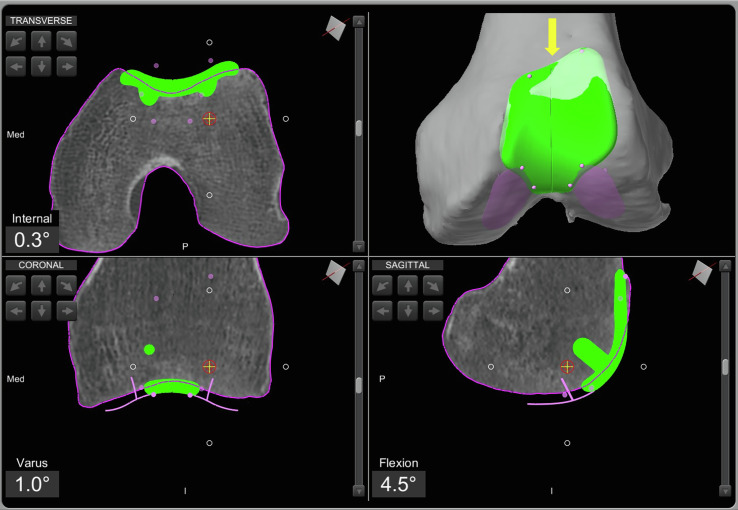
Patello-femoral arthroplasty preoperative planning using the Mako^®^ image-based robotic system (Stryker^®^, Mahwah, USA). The case is presented in Video 1.

At this stage, the position of the prosthetic implant, which will later be customized and adjusted during the intraoperative phase, is selected with the aim of achieving a match with the bone anatomy to create a resurfacing of the femoral trochlea (Figure 1).

### Step 2: Surgical approach and pins placement

A central incision is made, extending distally to the tibial insertion of the patellar tendon. Arthrotomy is performed using a medial mid-vastus approach. At this stage, it is essential to perform a cartilage check of all compartments.

The robotic technique is then employed using Mako^®^ robotic assistance (Stryker^®^, Mahwah, USA). Femoral pins are inserted through two anteromedial stab incisions at the mid-diaphysis of the femur. Optical arrays are positioned, and the hip center of rotation, bony landmarks, and cartilage thickness are recorded and matched with the preoperative CT model.

### Step 3: Trochlear mapping, intra-operative planning, and initial patellar tracking

A trochlear mapping is performed using a dedicated tool that does not invade the cartilage. Firstly, the trochlear groove is traced. Additionally, the cartilage surrounding the prosthetic implant area is mapped to determine its exact thickness.

Using the robotic software, intraoperative planning is completed by adjusting the positioning of the trochlear implant in all three planes: specifically, varus/valgus in the coronal plane, rotation in the axial plane, and flexion in the sagittal plane. This step is intended to refine the trochlear resurfacing, allowing the surgeon to match the patient’s trochlear groove with that of the implant, using a dedicated reference line displayed on the robotic system screen.

**Figure 2:**
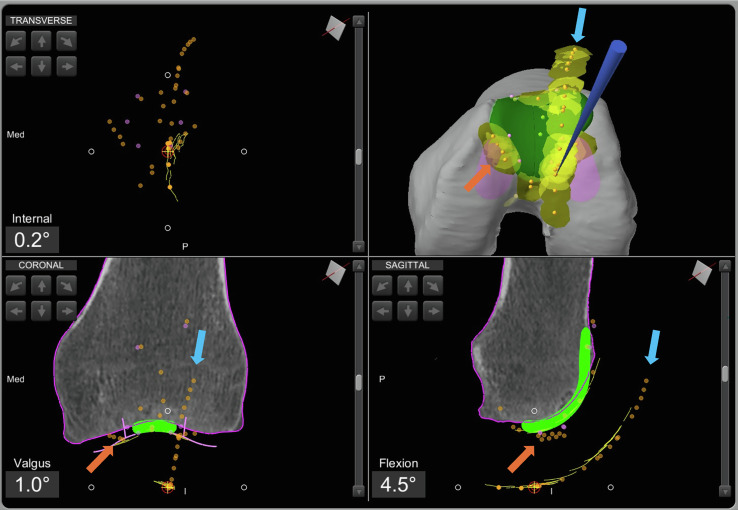
Intraoperative screenshot of patello-femoral arthroplasty intraoperative planning using Mako^®^ (Stryker^®^, Mahwah, USA). The blue arrow indicates the initial patellar tracking, while the orange arrow highlights the points of trochlear mapping.

A reference point is then identified at the center of the anterior patellar surface, and the initial patellar tracking is traced using a robotic tool. This tracking will later be used to guide patellar tracking correction during patellar resurfacing (Figure 2).

### Step 4: Bone preparation, trial component implant, and intraoperative patellar tracking

**Figure 3:**
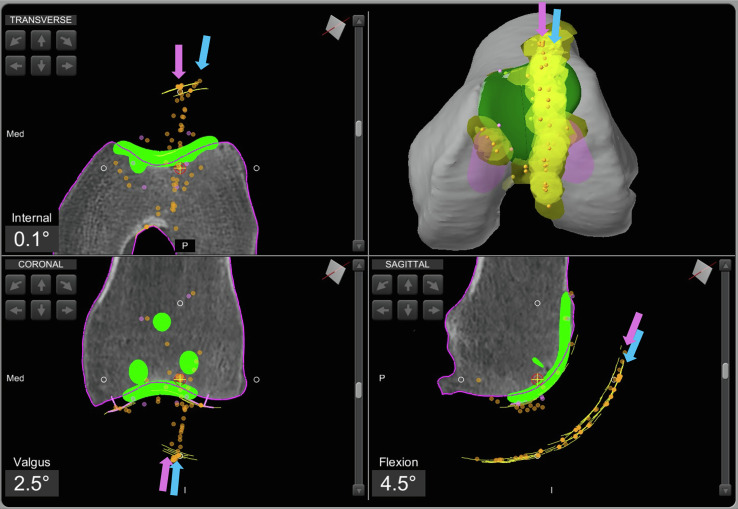
Intraoperative screenshot of patello-femoral arthroplasty using Mako^®^ (Stryker^®^, Mahwah, USA), with planning modified according to Functional Positioning principles. The blue arrow indicates the initial patellar tracking, while the violet arrow shows the tracking after placement of the trochlear trial component.

The bone is cut using the arm-assisted burr. The trial components are then positioned according to the sizing. At this stage, it is essential to ensure optimal trochlear resurfacing, avoiding any prosthetic overhang beyond the cartilage margins. Patellar tracking is then re-evaluated with the trial component in place (Figure 3).

### Step 5: Patellar cut and final patellar tracking

Based on intraoperative patellar tracking, the patellar cut is performed. At this stage, it is essential to ensure that the anterior offset is not increased and that patellar tracking is restored as close to neutral as possible.

These considerations guide the proper selection of the amount of patellar bone to be resected, with a minimum of 12 mm of residual bone thickness of the patella, aiming to restore the anterior compartment offset or slightly reduce it. During the preparation of the three peg holes for the patellar liner, the medio-lateral and antero-posterior positioning on the resected surface must be carefully assessed, to center patellar tracking relative to the trochlear component.

**Figure 4:**
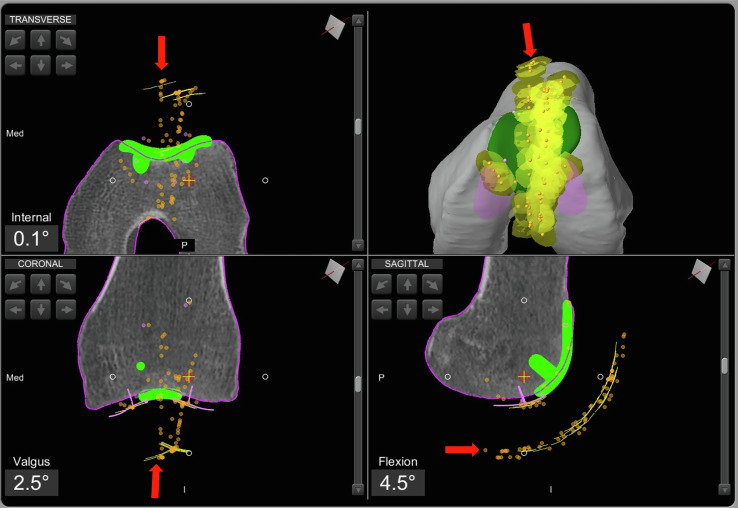
Intraoperative screenshot of patellofemoral arthroplasty using Mako^®^ (Stryker^®^, Mahwah, USA), with the red arrow indicating the final patellar tracking.

If necessary, a lateral and/or medial patellar facetectomy is performed. To confirm proper management of the anterior compartment, final patellar tracking is evaluated with the trial patellar liner in place (Figure 4).

### Step 6: Final implant cementation and closure

The cementation of the final components is carried out. Manual progressive compression is applied to the trochlear component, while a dedicated compression tool is used for the patella. Excess cement is then removed. After allowing sufficient time for the cement to set, capsular and skin closure are performed.

## Discussion

This article describes and illustrates the application of FP to PFA using an image-based robotic system. The role of FP, a three-dimensional alignment concept, is well established in TKA, while this article highlights its emerging application in small implants, particularly in PFA [12].

The success of PFA largely depends on appropriate patient selection. When either the surgical technique or the indications are suboptimal, PFA shows higher revision rates and lower implant survival compared to TKA [4, 11]. In the short term, complications are often related to implant misposition or patellar maltracking, which can result in painful instability, subluxation, or dislocation. In the long term, the most common cause of failure is the progression of tibiofemoral osteoarthritis (TF-OA) [2]. However, in cases of progressive TF-OA, it is possible to perform a staged addition of medial or lateral unicompartmental knee arthroplasty [13, 14].

The new generation of trochlea-friendly implant designs, which have demonstrated high satisfaction rates and low revision rates, allows for better preservation of the native trochlear anatomy [2]. Robotic technology enables precise resurfacing, either by replicating the previously mapped trochlear groove or by outlining the implant perimeter to position the final prosthesis according to resurfacing principles. Table 1 summarizes the main differences between the manual technique, imageless robotic, and image-based robotic approaches [2].

**Table 1:** Comparison between different techniques for patella-femoral arthroplasty: manual, robotic imageless, and robotic image-based.

	Manual technique	Imageless Robotic	Image-based Robotic
Planning Time	Intraoperative (and X-rays), based on experience	Intraoperative after mapping	Preoperative, refined intraoperatively
Bone resection guidance	Manual instruments	Robotic burr guided by bone morphing	Robotic burr guided by CT model
Implant positioning accuracy	Depends on surgeon’s experience	Improved via intraoperative 3D mapping	High accuracy with pre-op and intra-op guidance
Patellar tracking	Evaluated manually	Evaluated manually	Tracked in real-time with robotic assistance
Patellar tilt improvement	Moderate	Superior to manual	Superior to manual; comparable to imageless
Component customization	Limited	Multi-planar adjustment in surgery	Multi-planar adjustment + pre-op planning
Surgical precision	Standard	Enhanced	Highly precise
Learning curve	Lower (if experienced)	Moderate	Steeper (due to software + imaging integration)

In PFA, patellar management plays a central role. As is well known, overstuffing of the anterior compartment is associated with poorer functional outcomes [15, 16]. The use of a robotic tool that allows intraoperative assessment of patellar offset and translation relative to the trochlea enables a personalized approach to patellar resection [17, 18]. This technology improves the precision of both the resection thickness and the positioning of the patellar liner on the cut surface.

The previously described steps allow for intraoperative adjustment of the patellar cut, resulting in customized patellar tracking and anterior compartment balancing. This approach is consistent with the principles of FP while also respecting the native knee kinematics, particularly about the third space. Table 2 reports the main tips and tricks of this technique.

**Table 2:** Key tips and tricks for image-based robotic patellofemoral arthroplasty with functional knee positioning.

Step	Tips and tricks
Pre-operative planning	Use CT-based navigation for 3D modeling and precise implant positioning. Aim for anatomical resurfacing of the femoral trochlea.
Surgical approach and pins placement	Use medial mid-vastus approach for optimal exposure. Perform complete cartilage assessment of all compartments. Insert femoral pins through anteromedial stab incisions at mid-diaphysis.
Trochlear mapping, intra-operative planning, and initial patellar tracking	Use non-invasive mapping tool to trace trochlear groove and cartilage thickness. Adjust implant position in coronal (varus/valgus), axial (rotation), and sagittal (flexion) planes. Mark the anterior patellar center for reference in patellar tracking.
Bone preparation, trial component implant, and intraoperative patellar tracking	Use robotic arm-assisted burr for precision bone cuts. Avoid prosthetic overhang beyond cartilage margins. Reassess patellar tracking after trial components are in place.
Patellar cut and final patellar tracking	Leave at least 12 mm of residual patellar bone. Avoid increasing anterior offset; restore or slightly reduce it. Center peg holes medio-laterally and antero-posteriorly. Perform medial or lateral facetectomy if needed.
Final implant cementation and closure	Apply manual compression on trochlear component, tool-assisted on patella. Thoroughly remove excess cement. Close capsule and skin properly after cement sets.

Emerging research supports the idea that advances in personalization within knee arthroplasty may lead to better functional outcomes, greater patient satisfaction, and more natural knee kinematics. When it comes to applying these principles to PFA, however, the long-term durability of such approaches is still under investigation, highlighting the need for further high-quality research.

## Conclusions

This personalized approach, applicable to PFA and referred to as FP, represents a promising advancement in the management of PF-OA. The key steps of this surgical technique include trochlear resurfacing assisted by an image-based robotic system and the restoration of patellar tracking, following a step-by-step approach that is both effective and reproducible. Future studies will help refine FP strategies and further optimize outcomes in these patients.

## Supplementary material

The supplemental material is available at https://www.sicot-j.org/10.1051/sicotj/2025029/olm. *Video 1*. Step-by-step surgical technique video of functional positioning in robotic patello-femoral arthroplasty.
